# Stimulating immunoglobulin response by intramuscular delivery of exopolysaccharides-adjuvanted mannheimiosis vaccine in goats

**DOI:** 10.14202/vetworld.2022.2945-2952

**Published:** 2022-12-28

**Authors:** Ghaith Hussein Mansour, Laith Abdul Razzak, A. Suvik, Mohd Effendy Abd. Wahid

**Affiliations:** 1 Institute of Marine Biotechnology, Universiti Malaysia Terengganu, 21030 Terengganu, Malaysia; 2 Faculty of Fisheries and Food Sciences, Universiti Malaysia Terengganu, 21030 Terengganu, Malaysia; 3 Faculty of Science and Marine Environment Universiti Malaysia Terengganu, 21030 Terengganu, Malaysia

**Keywords:** adjuvant, immunoglobulin, *Mannheimia Haemolytica*, mannheimiosis, vaccine

## Abstract

**Background and Aim:**

Pneumonic mannheimiosis (PM) is a common respiratory bacterial disease among small ruminants. Despite numerous management methods, vaccination remains a suitable strategy to combat or reduce PM in goats and sheep. Thus, a study was conducted in Malaysia to evaluate the immunogenicity of exopolysaccharide-adjuvanted *Mannheimia* *haemolytica* A2 vaccine (EPS-MHA2) under laboratory and field conditions for its potential use as an efficient vaccine against PM.

**Materials and Methods:**

This study induced immunoglobulin (Ig) responses following intramuscular (IM) delivery of the EPS-MHA2 vaccine on 12 goats for about 7 months. Goats were divided into three groups, with three goats per group, and they were vaccinated intramuscularly as follows: Group 1 was vaccinated with an adjuvanted vaccine prepared from formalin-killed *M. haemolytica* serotypes A2 and EPS excipient; Group 2 was vaccinated with formalin-killed *M*. *haemolytica* seed only, whereas Group 3 was injected with phosphate-buffered saline (PBS) as the negative control. Measures of specific immunity included serum IgM, IgG, and IgA as well as bronchoalveolar lavage fluid secretory IgA and the size and number of the bronchus-associated lymphoid tissue (BALT).

**Results:**

From the 1^st^ day of vaccination, Groups 1 and 2 showed a significant (p < 0.05) increase in serum IgM, IgG, and IgA levels. However, the antibodies started to decline 5-week post-vaccination, indicating that the booster dose was necessary. On the second exposure to the same vaccine (booster), the level of antibodies showed a significant increase (p < 0.05), particularly IgG. All groups were challenged intratracheally by virulent MHA2 2 weeks after the decline of second antibodies on the administration of booster. All goats were euthanatized and necropsied 4-week post-challenge. The number and size of the BALT in Group 1 goats significantly increased compared with those in Group 2 and the unvaccinated control. Bacteriological parameters were evaluated, in which MHA2 was reisolated successfully from lung samples in Group 3. The IgA level produced by the group vaccinated with EPS-MHA2 was significantly (p < 0.001) higher than that the MHA2 vaccine and PBS groups. All data obtained were analyzed statistically using a one-way analysis of variance. The results indicate that IM injection of EPS-MHA2 vaccine significantly enhanced the immune response against MHA2.

**Conclusion:**

Therefore, the addition of EPS to MHA2 (EPS-MHA2 vaccine) can effectively protect goats from lethal mannheimiosis infection. Factors such as the ideal concentration of EPS should be further studied to verify its application potential as a vaccine adjuvant, and the extraction of EPS from different microalgae species should be further investigated. This study showed a novel and exciting set of data and a vaccination system, in which the suppressive effects of mannheimiosis may be further investigated.

## Introduction

Pneumonic mannheimiosis (PM) is a common respiratory bacterial disease among small ruminants worldwide, including Malaysia’s production animals [1]. PM causes substantial economic losses in calf breeding in the USA [2] and Europe [3]. In addition, PM was reported in infected cattle and buffaloes in Malaysia in the early 1800s, which resulted in financial losses caused by mortality, cost of care, and reduced productivity over life. Pneumonic mannheimiosis was due to a Gram-negative bacterium, namely, *Mannheimia haemolytica*. *Mannheimia* *haemolytica* can cause bacterial respiratory death in goats and sheep. Mastitis develops in camels and ewes. It has also been isolated from certain wild birds and cattle [4–7]. In particular, *M. haemolytica* causes bovine pneumonic pasteurellosis (older name) or mannheimiosis. In addition, PM is due to one of two strains of *M. haemolytica* designed as Type A serotype 1 in cattle and Type A serotype 2 in sheep and goats [8]. Mortality is due to the aggregation of fibrinous exudate and macrophages in the bronchioles. The disease can rapidly progress from mild to fatal [9]. Mannheimiosis remains a challenging problem in goats in Malaysia [10]. At present, vaccination is considered as a common and practical therapeutic application for controlling the spread of this infectious disease [11]. Despite numerous management methods, vaccination remains the best method to combat or reduce PM in goats and sheep. Although MHA2 vaccines are available on the market, it continues to spread and fail to build up immunity against MHA2 because of the low immunogenicity of the disease [12]. The use of adjuvants is a potential approach to improve PM vaccines. In particular, exopolysaccharides (EPS) as immune enhancers could be used in vaccine preparation. On the contrary, microalgae can synthesize a large amount of EPS and enhance immune responses (adjuvanticity) [13].

Improved adjuvant selection is a recent development that aims to enhance vaccine potency. Consequently, better vaccines for prophylaxis and treatment can be advanced through the creation of more effective and safer adjuvants. The role of EPS as an adjuvant vaccine candidate must be studied in developing a new-generation vaccine for controlling PM in goats. Moreover, with the advancement in biotechnology and comprehensive understanding of EPS agents combined with the knowledge of the host immune response, the production of antibodies against MHA2 through an adjuvanted vaccine would be advantageous under a variety of circumstances.

The previous studies on the same vaccine EPS-MHA2 injected intramuscularly in rats show strong results [14]. The need for a field study of mannheimiosis in goats is paramount; thus, a trial using this EPS-MHA2 vaccine was conducted on a goat farm in Terengganu, and the result is presented hereby.

The selection of a vaccine candidate adjuvant that can confer successful protection against virulent MHA2 has proven to be difficult over the years. This study aimed to check the potential of microalgal EPS as a vaccine candidate and its ability to enhance humoral and systemic immunity development against MHA2. It also aims to systematically investigate the immunogenicity of combined EPS-MHA2 adjuvanted vaccine under laboratory and field conditions for the potential use of a combined adjuvanted vaccine in protecting goats from lethal mannheimiosis infection.

## Materials and Methods

### Ethical approval

This study was approved by Universiti Malaysia Terengganu (UMT) Animal Ethics Committee with reference number UMT/JKEPHT/2019/1.

### Study period and location

The study was conducted from September 2020 to January 2021. During the study period of 18 weeks, experimental goats were housed in a cage at a goat farm located in Tok Jembal-Kuala Terengganu/Malaysia under standard housing conditions 12 hours light and 12 hours dark cycle in an environmental temperature (36°C).

### Study animals

Katjang hybrid goats without any vaccination history against mannheimiosis were selected for this experiment. Nine clinically healthy male goats aged 5–6 months were purchased and numbered with animal tags. Goats were individually examined on reaching the goats’ farm. The study was started after the goat’s adaptation period (2 weeks). General health state, including body condition and clinical signs such as nasal discharge, coughing, weakness, and fever, was regularly evaluated from the 1^st^ day of purchasing.

### Vaccination experimental design

In three experimental groups, goats were randomly allocated into pens, three goats in each group under similar environmental conditions, and housed in ventilated pens at a goat farm in Toq jambal, Kuala Terengganu. Before the experiments, goats were fed a commercial pellet of 250 g/goat/day and supplemented with grass, and clean drinking water was given *ad libitum*.

Goats were divided into three groups, with three goats per group. All goats were vaccinated intramuscularly: Group 1 was vaccinated with an adjuvanted vaccine prepared from formalin-killed *M. haemolytica* serotypes A2 and EPS excipient; Group 2 was vaccinated with formalin-killed *M*. *haemolytica* seed only, whereas Group 3 was injected with phosphate-buffered saline (PBS) as the negative control. In goat serum samples, immunoglobulin (Ig) (IgM, IgG, and IgA) concentration was calculated by enzyme-linked immunosorbent assay (ELISA).

By the end of the study period, all goats were slaughtered humanely, and the lungs were examined histologically with regard to the size (mm^2^) of bronchial-associated lymphoid tissue (BALT) and the number of lymphocytes infiltrated within the bronchial area [1].

### Preparation of experimental adjuvant and vaccines

The vaccines for this experiment were prepared at the IMB microbiology laboratory, UMT, Malaysia. Based on the highest isolation of goats from various places in Malaysia, MHA2 was selected and used as a candidate vaccine strain [10, 15, 16]. The *M. haemolytica* serotype A2 was stored at the IMB germ bank in deep freezing at −80°C. Using tryptic soy broth, *M. haemolytica* was cultured and grown. The tubes used for culturing underwent a sterility test, in which the tubes incubated for 48 h were checked for contamination before using it, and the tubes with no growth were used. Afterward, the tubes were sub-cultured and incubated at 37°C for 16 h into the broth. The presence of turbidity indicated growth. Then, *M. haemolytica* was cultured on blood agar. Colony morphology, purity, hemolysis pattern, and Gram stain results were primarily identified. Vitek 2^®^ System compact (BioMériuex SA, USA) was used for identification. Culture plates were grown at 37°C in a rotatory shaking incubator for 16 h [17, 18]. About 30 colonies of pure bacteria were transferred into 250 mL of brain heart infusion broth (Oxoid). Then, the culture was shaken using a shaker incubator (Labwit Scientific, Australia) and incubated at 37°C for 18 h for replications. After incubation, serial dilutions and standard plate counts were used to measure the culture concentration. Then, *M. haemolytica* cells were killed by adding 1% of buffered formalin (BF, 0.25 mL/25 mL) (Sigma, USA) in PBS and incubated overnight at 4°C. Next, the bacterial cells were washed 5 times using PBS (pH 7.4) and centrifuged at 6000× *g* for 15 min using a refrigerated centrifuge CF16RXII (Hitachi Koki Co. Ltd., Japan) to eliminate the remaining formalin from the cultures. The bacteria were washed 5 times again with sterile PBS, and the concentration was adjusted to 10^6^ Colony-forming unit (CFU)/mL using an optical density.

### Construction of adjuvanted vaccine (EPS-MHA2 vaccine)

One milligram of extracted *Tetraselmis chui* EPS powder was extracted [9, 19] and diluted in 100 mL of PBS to prepare a stock solution with a final concentration of 0.01 mg/mL. Then, only 50 μL of diluted EPS was combined with the pellet of 10^6^ CFU/mL of formalin-killed MHA2 vaccine to make EPS-MHA2 vaccine [20].

### Challenge infection

All goats at week 14 (7 weeks after the second vaccination at week 7) were challenged with live MHA2 at 10^8^ CFU/mL through intratracheal injection. At week 18, 4 weeks after the challenge, all goats from each group were euthanized in accordance with the animal ethics code to collect the right apical lobe for histological testing and serum for serological testing [16].

### Isolation of *M. haemolytica*

At the time of euthanasia, the heart, lungs, liver, and blood were collected. For *M. haemolytica* isolation, samples from the specimens were processed. Samples from which *M. haemolytica* could not be isolated until it was recultured up to 3 times were considered harmful and discarded. The potential representative colony was recognized as *M. haemolytica* by Vitek 2^®^ System compact (BioMériuex SA). No growth plates were reincubated for 48 h and discarded if such plates were negative.

### Determination of antibody titers

Blood samples (3 mL) were obtained weekly from each goat by jugular vein for immunogenicity testing. Afterward, the samples were left to clot at room temperature (27°C) and then centrifuged for 10 min at 8000× *g*. Then, the serum was removed to a new cap tube and processed at −30°C. ELISA was used for immunogenicity evaluation.

### Enzyme-linked immunosorbent assay titer

In goat serum samples, Ig (IgM, IgG, and IgA) concentration was calculated by using an indirect ELISA titer, as previously described by Effendy *et al*. [16]. Plates were coated with MHA2 suspension and the remaining protein-binding sites in the coated wells were blocked using a blocking buffer. Next, goat serum was added. The detection agent, rabbit anti-goat IgM horseradish peroxidase conjugate (Bethyl, USA), rabbit anti-goat IgG horseradish peroxidase conjugate (Santa Cruz Biotechnology, USA), and rabbit anti-goat IgA horseradish peroxidase conjugate (Abcam, USA) for serum samples of goat and lung lavage of goat were used as secondary antibodies and added last. At room temperature, each step was incubated for an hour and finished by inverting the plates, shaking the solution in the wells, and removing unbound materials by washing the plate. Finally, a chromogenic agent was added and incubated for 30 min at 37°C, and the plate was read using an ELISA microplate reader (Thermo-Scientific Multiskan Ascent, TM, USA) at an absorbance of 450 nM.

### Lung lavage collection and histopathological assessment

All goats were euthanized 4-week post-challenge. The autopsy was performed on all goats and the lungs were collected for microscopic examination. Lungs were removed without perfusion from the thorax *en bloc*. Lung lavage was collected by adding sterile PBS (pH 7.4) into the trachea through a 500-mL sterile dispensing bottle. The lung was gently massaged for 30 s before lung lavage was withdrawn and kept in a sterile Falcon™ tube. Then, the lungs were inflated with 10% of BF through a tracheal cannula. The lungs and other tissues were preserved for 24–72 h in 10% of Neutral buffered formalin before being cut for paraffin embedding. The lungs were trimmed along the edges of the right apical lobe. At 5 mm, paraffin-embedded tissues were made and stained with hematoxylin and eosin for histological examinations.

### Statistical analysis

In evaluating the titers, repetitive measure analysis of variance was used, and a Tukey was used to allow pair comparisons between vaccination intervals and groups. Using the Student’s t-measure, antibody titers against MHA2 were evaluated. The findings were evaluated using GraphPad Prism, version 8.0 (GraphPad Software Inc, San Diego, CA, USA), and p < 0.05 was considered significant.

## Results

### Clinical observations

All challenged goats coughed moderately during the intratracheal challenge injection. Throughout the first 3-day post-challenge, none of the challenged goats died, but 45% (n = 4) of them showed clinical signs of respiratory distress, such as mild-to-moderate sneezing and coughing.

### Serological IgM, IgM, and IgA response

**Figure-1:**
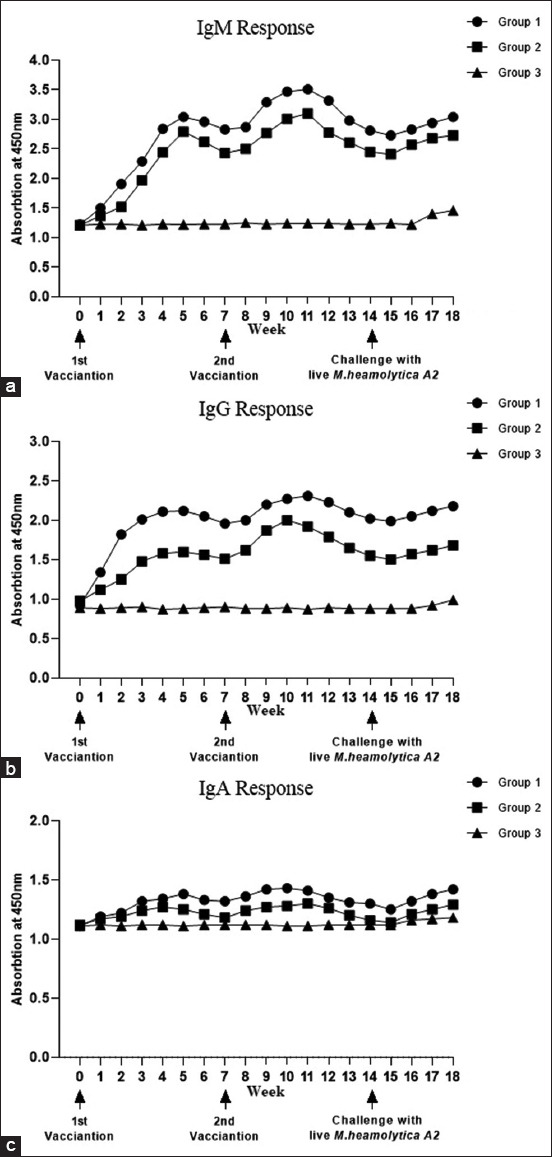
Serum IgM, IgG, and IgA (a, b, and c patches respectively) antibody response levels in goats following intramuscular vaccination. Notice that Exopolysaccharide*-Mannheimia haemolytica* A2 vaccine Group (1), *M. haemolytica* A2 vaccine Group (2), and phosphate buffer saline Group (3). A one-way analysis of variance was used, followed by Tukey’s multiple comparisons tests. Ig=Immunoglobulin.

Before vaccination, all goats had low serum IgG, IgM, and IgA levels against MHA2. However, after the first intramuscular (IM) vaccination, the IgG response of goats in Group 1 and Group 2 increased significantly (p < 0.05) as compared with that in control Group 3 (Figure-1).

Serum IgG and IgM levels were high as early as week one post-vaccination, and they started to decrease by week 5, although they have remained significantly higher (p < 0.05) than the control. Following the IM administration of the booster dose 2 weeks after it started to decrease, the IgM and IgG levels in Group 1 and Group 2 increased significantly (p < 0.05) compared with those in control Group 3. At the time of challenge with wild-type MHA2, the serum levels of the vaccinated and control groups increased significantly (p < 0.05). The IgG levels of vaccinated Group 1 were significantly (p < 0.05) higher than those of Group 2 and Group 3 post-challenge.

In general, the serum IgM, IgG, and IgA levels at week 6 post-vaccination were significantly lower (p < 0.05) than those at week 12 post-second-vaccination (booster). The serum IgM, IgG, and IgA levels in vaccinated goats in Group 1 were significantly higher (p < 0.05) than those in the control group and Group 2 throughout the 18-week study period of vaccination (Figure-1).

### Bacterial isolations

*Mannheimia haemolytica* was successfully isolated from the lungs of goats who were slaughtered humanely 4 weeks after the challenge. Under a microscope, the bacteria positively showed a Gram-negative property for Gram stain. Vitek 2^®^ System compact (BioMériuex SA) was used to identify the types of bacteria. Isolation was made from the lungs of only n = 1 (33%) vaccinated goats in Group 2 and n = 3 (100%) of the control goats in Group 3. However, none (0%) of the vaccinated goats in Group 1 had *M. haemolytica* in their lungs.

### Histology of the BALT

The lung of the right apical lobe, the first route of lung exposure to pathogens, was selected and processed for histology from all groups. Four weeks after the challenge, all groups had their complete right apical lobe lung sectioned at 5 μm, and the paraffin sections were stained with hematoxylin and eosin. Based on histological examination, the five-site formation of BALT development around the bronchus and bronchi was selected to detect the BALT and categorize it as either nodular or aggregate. The stained slide was placed on a light microscope (×600, SM-LUX, Leitz, Wetzlar, Germany) and viewed using 5× and 20× objective lenses. The surface area and perimeter of aggregate and modular BALT were measured using a 20× objective lens. The perimeter surrounding the BALT was created using the polygon function of system software. Right-clicking on the mouse yielded the measurements of the surface area (μm^2^) and lymphocyte number of the observed BALT. The lymphocytes within BALT were manually counted using the counting function of system software.

**Figure-2:**
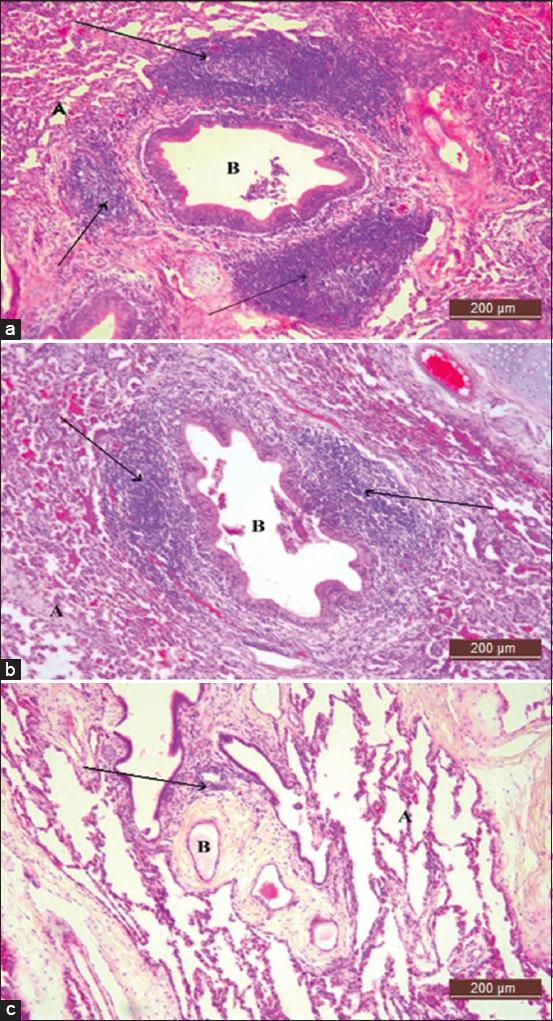
Photomicrograph of lymphoid aggregates (arrow) that were stained with Hematoxylin and Eosin (H & E) and formed in the lung of goats treated with (a) exopolysaccharide*-Mannheimia haemolytica* A2 vaccine Group 1, (b) *M. haemolytica* A2 vaccine Group 2, and (c) PBS Group 3. A: Alveolar space; B: Bronchiole. Type of formation nodular and aggregates. Total magnification 4× and 40×.

The dark purplish-blue color indicated the development of BALT on lung tissues and the structure of BALT, which was observed around the wall of the bronchus and bronchioles, categorized as aggregates and nodular BALT (Figure-2). The area of the bronchus or bronchioles was surrounded with aggregations of lymphocytes without suppression and the mucosal epithelium was categorized as an aggregate type of BALT, which was always associated with the bifurcation of the major bronchus [16]. No inflammation (edema or fibrin) was detected in the lung tissue in vaccinated goats. The PBS group showed a severe lesion of pneumonic pasteurellosis with thickened interalveolar space. The other two groups showed a less histological lesion of pneumonic pasteurellosis; meanwhile, the EPS-MHA2 vaccine group showed more formation of BALT than the other groups. The formation types included nodular and aggregate.

### Size and lymphocyte number of BALT

**Figure-3:**
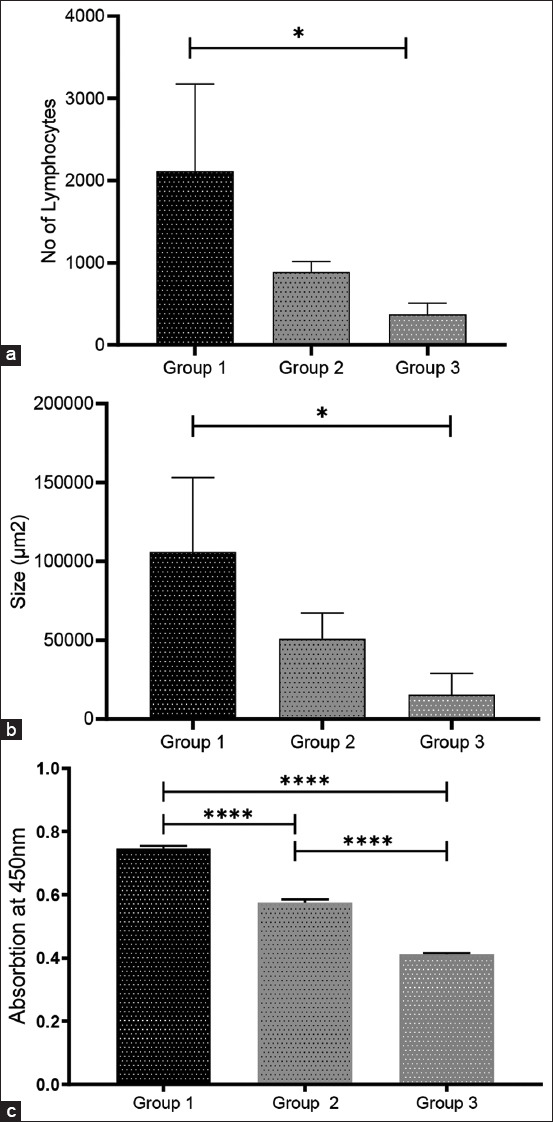
(a) Comparison of the size of BALT area between PBS: Phosphate Buffered Saline group, MHA2: *Mannheimia haemolytica* A2 vaccine group and EPS-MHA2: Exopolysaccharide-*M. haemolytica* A2 vaccine group. (b) Comparison of the number of lymphocytes between PBS: Phosphate-buffered saline Group 3, MHA2: *Mannheimia haemolytica* A2 vaccine Group 2, and EPS-MHA2: EPS-*M. haemolytica* A2 vaccine Group 1 that occurred in the BALT area. (c) The development pattern of IgA response in the lung lavage fluid of goats was collected at the end of 4 weeks of intratracheal exposure. Value means ± SEM, (n = 3) goats per group; *p < 0.05, ****p > 0.001. One-way analysis of variance was performed, followed by Tukey’s multiple tests for analysis.

Apart from reviewing the histological lesion in lung tissues, the differences in BALT responses should be highlighted by calculating the average size area (μm^2^) of BALT formed and the number of lymphocytes formed within BALT using the LAS 4.0 system (Leica Microsystems, Teban Gardens, Singapore). As shown in Figure-3a, the average size of BALT showed an increasing size in the EPS-MHA2 vaccine group (124854 μm^2^), MHA2 vaccine group (65462 μm^2^), and PBS group (15411 μm^2^). However, when comparing the average size of BALT formed among the groups, the group that was vaccinated with MHA2 was significantly higher (p < 0.05) compared with the PBS group; meanwhile, the EPS-MHA2 vaccine group showed a significantly high (p < 0.05) average BALT size compared with the MHA2 vaccine and PBS groups.

However, the EPS-MHA2 vaccine group is still regarded as the highest in BALT formation when compared with the other two groups. As shown in Figure-3b, the developmental pattern seems to differ in the average number of lymphocytes. Only the EPS-MHA2 vaccine group (4385) showed a significantly (p < 0.05) high average number of lymphocytes when compared with the MHA2 vaccine group (892) and PBS group (372).

The average size of individual BALT, or the general area occupied by numerous BLAT, was calculated to highlight the variations in inflammatory responses generated in the lung tissue of vaccinated/unvaccinated goats 4 weeks after the infection challenge. The average size of whole lymphoid BALT was significantly larger in the lungs of goats vaccinated with challenged EPS-MHA2 (124854 μm^2^) than in goats vaccinated only with MHA2 (65462 μm^2^) and PBS (15411 μm^2^). Differences were considered significant at p > 0.05 (Figure-3a). Bronchus-associated lymphoid tissue also showed a significantly (p < 0.05) high average number of lymphocytes when compared with the MHA2 vaccine group (892) and PBS group (372). The total area and number of lymphocytes occupied by BALT were larger in Group 1 as compared with those in Group 2. However, these differences were not statistically significant (Figures-3a and b). On the contrary, the level of secretory IgA (sIgA) in the lung lavage produced from the group vaccinated with EPS-MH was significantly higher (p < 0.05) when compared with the MH vaccine and PBS groups (Figure-3c).

## Discussion

This study used microalgal EPS as a potential adjuvant when delivered with MHA2 seeds. On a previous work on rats, a high percentage of rats vaccinated with EPS-MHA2 were significantly protected against lethal mannheimiosis infection compared with the control groups, and these rats presented well-organized BALT in their lungs [14]. The vaccine candidate was given intramuscularly, which is a desirable option over traditional parenteral formulations for respiratory infections. Nevertheless, few studies have shown adequate protection after administering the polysaccharide-based vaccine [21, 22]. Commercial vaccines against *M*. *haemolytica* serotypes include live-attenuated leukotoxin, capsule, and subunit vaccines, as well as sodium salicylate extract and potassium thiocyanate [12, 23, 24]. Most of the aforementioned vaccines are poorly immunogenic and antigenic and they cannot cross-protect against infections with other *M*. *haemolytica* serotypes [25].

Following IM immunization of goats, the microalgal recombinant EPS-MHA2 vaccine significantly improved their protective capacity against mannheimiosis. The high level of protection observed was associated with progressive BALT formation and a high level of specific antibodies in sera and lavage IgA antibody-vaccinated goats. This result was contrary to that of the control groups. Serum from goats vaccinated with EPS-MHA2 showed an increased production of antigen-specific antibodies, compared with the antibody levels in sera from the MHA2- and PBS-immunized groups, which presented low levels of Ig. This result is due to the fact that EPS increases local recruitment, antigen processing, and presentation efficiency of antigen-presenting cells at the vaccination site, and the proliferation of antigen-specific T cells and antibody-secreting B cells migrates to distant effector sites such as the lung mucosal membrane [26]. Data from the study reveal that goats have significant antigen-specific antibodies, which correspond with protection against challenge. In addition, these antibodies have a high affinity, which is associated with the protective immune response observed without a trace of the bacteria in the lung of the EPS-MHA2-vaccinated group. However, following the challenge, antibodies are generated and promptly destroyed locally, indicating infection’s confinement in the lungs.

Moreover, the formation of organized lymphoid structures in the bronchial of the lungs following IM immunization is described in this work. In a previous report, the morphologic and hyperplastic changes in the BALT resulted from the stimulation of antigenic substances through vaccination or lung infection. This result correlates with this finding of the significant values of the average size of BALT and the number of lymphocytes. The group vaccinated with EPS-MHA2 vaccine showed a significant (p < 0.05) increase in the size of BALT area and in the number of lymphocytes within BALT when compared with the MHA2 and PBS groups. This observation indicated that the local immune responses were successfully stimulated following EPS-MHA2 vaccination, which was regarded as antigenic stimulation for the formation of lymphoid aggregates at the bronchus, thereby producing the mucosal secretory antibody that protects the mucosal membrane of the lungs from further colonization of MHA2 [16, 27–29].

Another remarkable result was the excellent stability of BALT, which was observed at later periods following intratracheal exposure. This result is contrary to the limited number of lymphoid aggregates found in the lungs of the control group. This phenomenon is due to the fact that protein and peptide antigens can linger in these regions for long periods, activating B and T cells and maintaining local antibodies [30, 31]. In addition, the spleens and livers from immunized and subsequently challenged goats also did not contain any viable MHA2. Therefore, immune responses that begin in the lungs can also regulate disseminated illness, resulting in bacterial clearance from peripheral organs.

Secretory IgA was developed in the lungs among the groups of goats. Based on the study conducted by Effendy *et al*. [16], high levels of sIgA were found in lung lavage fluid after exposure to whole-cell formalin-killed *Pasteurella haemolytica* A2 by intranasal inhalation in this study. According to Eng *et al*. [32], PCEP adjuvants could also increase sIgA antigen-specific titers in the lungs of mice following intranasal immunization. Therefore, using EPS as an adjuvant for the MHA2 vaccine could strengthen mucosal responses by producing significant levels of sIgA in the lung mucosa. Moreover, this production demonstrated the significance of sIgA as the principal mucosal antibody of the mucosal immune system, which provides robust defense against a vast range of pathogens at the mucosal surfaces of the lung through immunological intracellular neutralization, excretion, and antigen exclusion [33, 34].

Therefore, this experiment could verify whether the use of an adjuvanted vaccine will generate high antibodies against MHA2 and protect vaccinated goats from challenge with live MHA2. The observation and study of EPS, which acquires immunostimulatory activity and increases the immune response [35, 36], created the hypothesis in this study.

## Conclusion

When EPS is combined with an antigen in the vaccine, significant protection can be obtained. Using EPS as an adjuvant allows for the condensing and standardization of particular antigen content in vaccination doses. In this study, the findings on EPS vaccination as an adjuvant in goats can serve as a basis for developing similar *M*. *haemolytica* vaccines. Taken together, these findings provide the rationale for pursuing the continuous invention of vaccines to treat and prevent mannheimiosis disease. The findings of this study have shown that EPS is an efficient adjuvant which can be used for subunit vaccine production. However, the potential application of EPS as an adjuvant should be further studied.

## Authors’ Contributions

GHM, LAR, AS, and MEAW: Conceived and designed the experiments. GHM, LAR, and MEAW: Performed the experiments. LAR and MEAW: Analyzed and interpreted the data. GHM and MEAW: Statistical analysis. MEAW: Contributed reagents, materials, and analysis tools. GHM, MEAW, and LAR: Wrote the manuscript. All authors have read and approved the final manuscript.
